# Sensory-Evoked 40-Hz Gamma Oscillation Improves Sleep and Daily Living Activities in Alzheimer’s Disease Patients

**DOI:** 10.3389/fnsys.2021.746859

**Published:** 2021-09-24

**Authors:** Aylin Cimenser, Evan Hempel, Taylor Travers, Nathan Strozewski, Karen Martin, Zach Malchano, Mihály Hajós

**Affiliations:** ^1^Cognito Therapeutics, Inc., Cambridge, MA, United States; ^2^Department of Comparative Medicine, Yale University School of Medicine, New Haven, CT, United States

**Keywords:** Alzheimer’s disease, sleep, actigraphy, sensory-evoked gamma oscillation, activities of daily living

## Abstract

Pathological proteins contributing to Alzheimer’s disease (AD) are known to disrupt normal neuronal functions in the brain, leading to unbalanced neuronal excitatory-inhibitory tone, distorted neuronal synchrony, and network oscillations. However, it has been proposed that abnormalities in neuronal activity directly contribute to the pathogenesis of the disease, and in fact it has been demonstrated that induction of synchronized 40 Hz gamma oscillation of neuronal networks by sensory stimulation reverses AD-related pathological markers in transgenic mice carrying AD-related human pathological genes. Based on these findings, the current study evaluated whether non-invasive sensory stimulation inducing cortical 40 Hz gamma oscillation is clinically beneficial for AD patients. Patients with mild to moderate AD (*n* = 22) were randomized to active treatment group (*n* = 14; gamma sensory stimulation therapy) or to sham group (*n* = 8). Participants in the active treatment group received precisely timed, 40 Hz visual and auditory stimulations during eye-closed condition to induce cortical 40 Hz steady-state oscillations in 1-h daily sessions over a 6-month period. Participants in the sham group were exposed to similar sensory stimulation designed to not evoke cortical 40 Hz steady-state oscillations that are observed in the active treatment patients. During the trial, nighttime activities of the patients were monitored with continuous actigraphy recordings, and their functional abilities were measured by Alzheimer’s Disease Cooperative Study – Activities of Daily Living (ADCS-ADL) scale. Results of this study demonstrated that 1-h daily therapy was well tolerated throughout the 6-month treatment period by all subjects. Patients receiving gamma sensory stimulation showed significantly reduced nighttime active periods, in contrast, to deterioration in sleep quality in sham group patients. Patients in the sham group also showed the expected, significant decline in ADCS-ADL scores, whereas patients in the gamma sensory stimulation group fully maintained their functional abilities over the 6-month period. These findings confirm the safe application of 40 Hz sensory stimulation in AD patients and demonstrate a high adherence to daily treatment. Furthermore, this is the first time that beneficial clinical effects of the therapy are reported, justifying expanded and longer trials to explore additional clinical benefits and disease-modifying properties of gamma sensory stimulation therapy.

**Clinical Trial Registration:** clinicaltrials.gov, identifier: NCT03556280.

## Introduction

Despite significant advances in our understanding of pathological mechanisms involved in Alzheimer’s disease (AD), treatment options are still limited. AD is a progressive neurodegenerative illness with long preclinical and prodromal phases, resulting in cognitive dysfunction, behavioral abnormalities, and impaired performance of activity of daily living (Masters et al., 2015). It has been well-established that hallmarks of AD-related pathological proteins, including amyloid-β (Aβ) oligomers and hyperphosphorylated tau, disrupt normal neuronal functions in the brain, leading to unbalanced neuronal excitatory-inhibitory tone, distorted neuronal synchrony, and network oscillations (Mucke and Selkoe, 2012; Das et al., 2018). However, the probability that abnormal neuronal activity, such as hyperexcitability directly contributes to the pathogenesis of AD has recently been proposed (Canter et al., 2016; Hijazi et al., 2020). Remarkably, it has been recently demonstrated that induction of synchronized gamma oscillation of neuronal networks by optogenetic or sensory stimulation effectively halts or reverses AD-related pathology in transgenic mice carrying AD-related human pathological genes (Iaccarino et al., 2016; Adaikkan et al., 2019; Martorell et al., 2019; Zhang et al., 2020). Several mechanisms downstream to gamma-oscillations have been identified, such as activation of brain innate immune system leading to increased phagocytic activity of microglia, together with augmented vasodilation and transcytosis across the brain endothelium contributing to the removal of pathological proteins. Transgenic animals sub-chronically exposed to 40 Hz sensory stimulation also showed reduced neurodegeneration and brain atrophy, increased synaptic density, improved cognitive function, and normalized circadian rhythm (Adaikkan et al., 2019; Martorell et al., 2019; Yao et al., 2020).

**FIGURE 1:**
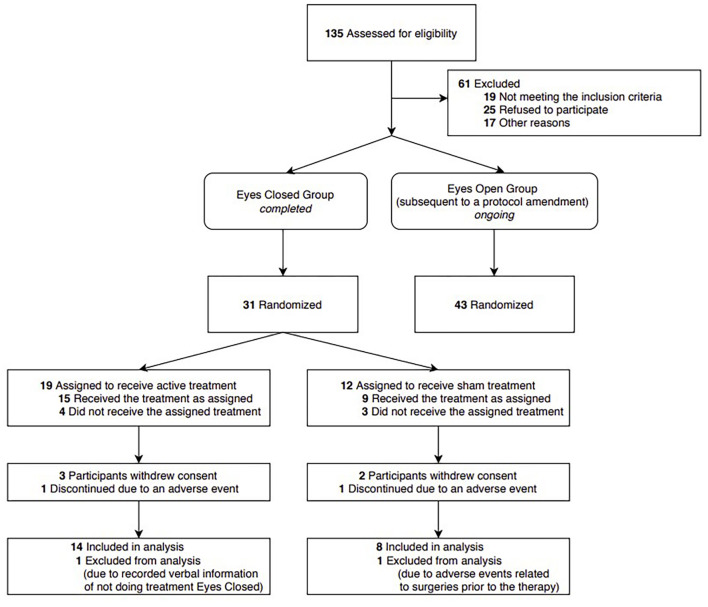
CONSORT flow diagram and study design. Current analysis included patients receiving treatment during eyes-closed condition and completed the 6-month trial, including all patients in the treatment and sham groups. Patients not included in this report are currently receiving therapy during the eyes-open condition in the on-going trial.

Based on these experimental findings, current clinical trials are evaluating the safety and efficacy of 40 Hz audio-visual stimulation in AD patients. The present study analyzed clinical results of participants of the clinical trial Overture (NCT03556280) who received treatment during eye-closed conditions and completed the 6-month trial period (Figure 1). We report here safety and adherence findings together with results of nighttime activities obtained by continuous monitoring with actigraphy, and functional abilities assessed by Alzheimer’s Disease Cooperative Study Activities of Daily Living (ADCS-ADL) scale (Galasko et al., 1997).

Analysis of nighttime activities has been a key importance of this study. Recent experimental findings demonstrated that 40 Hz light stimulation over 30 days period significantly reduced abnormally high activity in Aβ overproducing transgenic (APP/PS1) mice during their rest phase and normalized hypothalamic central clock function (Yao et al., 2020). Sleep disorders are more frequent and more severe in mild cognitive impairment (MCI) and AD patients compared to cognitively normal older adults (Mander et al., 2015). A cardinal complaint about sleep of these patients is excessive nocturnal awakenings. Therefore, one of the aims of our trial was to evaluate the applicability of actigraphy recordings of nighttime activities of AD patients over a long-time period, monitoring potential benefits of therapeutic intervention on sleep quality. Establishing a relatively easy and practical method for monitoring nighttime activity can be highly valuable, since accumulating clinical data demonstrate a strong, bidirectional connection between sleep disorders and disease progression in AD, indicating a vicious circle contributing to AD progression (Bubu et al., 2020; Wang and Holtzman, 2020).

In the present study, nighttime activity has been monitored daily throughout the entire 6-month study period with a wrist-worn actigraphy watch, using this data, we identified and measured durations of active periods in sleep. Although actigraphy can detect rest and active periods, cross-validation with polysomnography sleep studies has led to the generally accepted interpretation that periods of nighttime restfulness are considered a reasonable measure of sleep (Tachikawa et al., 2017; Smith et al., 2018). Furthermore, previous studies have demonstrated that for AD patients, assessment of sleep by actigraphy is more accurate and reliable than a subjective report of sleep quality due to the ease of use, passive effort, and continuous measurement (Canazei et al., 2019) and has been more directly linked to cognitive impairment (Cabanel et al., 2020). In line with these observations, recent experimental and epidemiological findings demonstrate that sleep disorders represent a risk for developing AD, and a close correlation exists between sleep abnormalities and decline in activities of daily living of AD patients (Macedo et al., 2017; Hennawy et al., 2019; Bubu et al., 2020). Therefore, in addition of monitoring nighttime activities, functional abilities of patients were assessed by the (ADCS-ADL) scale (Galasko et al., 1997).

## Materials and Methods

### Patients and Study Design

The objective of the present study was to evaluate the effects of daily, 1-h 40 Hz sensory stimulation over a 6-month period on sleep quality and daily living activities in mild to moderate AD patients. Patients included in this analysis formed a subgroup of patients enrolled in, and completed an ongoing Phase I/II randomized, controlled single-blind clinical trial^1^. Patients gave written informed consent, and they were randomized to treatment or sham groups (Figure 1, CONSORT Flow Diagram and Study Design).

Patients included in the present analysis (*n* = 22) were clinically diagnosed having mild to moderate AD and were under the care of their care neurologist. Inclusion criteria were age of 55 years or older, Mini-Mental State Examination (MMSE) score 14–26 and participation of a caregiver, whereas exclusion criteria included profound hearing or visual impairment, seizure disorder, or implantable, non-MR compatible devices. Patients on AD medication therapy with acetylcholine esterase inhibitors (donepezil, rivastigmine) could enroll, but their dosing were maintained the same during the trial. Use of memantine was not permitted during the study. Participants were asked about their medications every 4 weeks. In the treatment group 50% of participants and the sham group 75% of participants reported using donepezil during the study. One participant in the treatment group reported using rivastigmine. Among the participants who were studied in the sleep analysis, only one participant reported using sleep medication. This participant was in the sham group and reported using melatonin. Demographic and clinical characteristics of patients are given in Table 1. Patients in active treatment group (*n* = 14; Gamma sensory stimulation therapy) received precisely timed, 40 Hz visual and auditory stimulations during eye-closed condition to induce cortical 40 Hz steady-state oscillations in 1-h daily sessions over a 6-month period. Patients in the sham group (*n* = 8) were exposed to sensory stimulation at a high frequency that has been previously shown to not evoke cortical steady-state oscillations at stimulus frequency (Herrmann, 2001). Patients were not required to do the treatment at the same time but were encouraged to do it in the morning hours. All 22 patients completed the 6-month trial.

**TABLE 1:** Demographic and clinical characteristics of patients at the initial assessment.

Characteristic	Treatment group (*N* = 14)	Sham group (*N* = 8)
**Demographics**		
Age in years, mean ± SD	66.5 ± 8.0	73.5 ± 6.6
Gender, no (%)		
Female	10 (71)	5 (63)
Male	4 (29)	3 (37)
Race and Ethnicity, no (%)		
White	14 (100)	8 (100)
Hispanic or Latino	1 (7)	0 (0)
**APOE4**–**status, no** (%)		
Non-carrier	5 (36)	3 (37.5)
Heterozygous	7 (50)	4 (50)
Homozygous	1 (7)	1 (12.5)
Ambiguous*	1 (7)	NONE
**Cognitive assessment**		
MMSE score†, mean ± SD	19.9 ± 2.8	18.5 ± 2.7
**Functional assessment**		
ADCS-ADL score 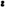 , mean ± SD	61.8 ± 9.1	65.0 ± 10.4

*Gs270: [rs429358(C; T), rs7412(C; T)]. Likely lo be heterozygous (APOE E2/E4) but non-carrier (APOE4 E1/E3) possibility exits.

*†Mini-Mental State Examination (MMSE) scores range between 0 and 30, higher scores indicating better cognitive performance.*

*
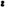
Alzheimer’s Disease Cooperative Study – Activities of Daily Living (ADCS-ADL) scores range between 0 and 78, higher scores indicating better functioning.*

### Gamma Sensory Stimulation Device

The gamma sensory stimulation device is a therapeutic system developed by Cognito Therapeutics, Inc., that includes a handheld controller, an eye-set for visual stimulation, and headphones for auditory stimulation, that work together to deliver precisely timed, non-invasive 40 Hz stimulation to induce steady-state gamma brainwave activity. Stimulation output parameters are determined and verified by a physician based on both patient-reported comfort information and in-clinic electroencephalography (EEG) evaluation. The Gamma sensory stimulation device is then configured to the determined settings, and all subsequent use is within this predefined operating range. Once prescribed, the patient uses the device at home for daily sessions lasting 1 h. The on-off periods of the visual stimulation are perceivable by the patient but not disruptive; the patient remains aware of their surroundings and can converse with a care partner during use of the system. The patient can adjust the brightness of the visual stimulation and the volume of the auditory stimulation within this predefined operating range via push buttons on the controller, with assistance from a care partner as needed. The system logs device usage and stimulation output settings for adherence monitoring; this information is uploaded to a secure cloud server for remote monitoring.

### Monitoring Sleep Restful and Active Periods With Actigraphy and Signal Processing

Effects of gamma sensory stimulation therapy on durations of restful and active periods during sleep were determined over a 6-month period by continuous monitoring activity of AD patients with a wrist worn actigraphy watch (ActiGraph GT9X), recording 3-axis acceleration at 30 Hz sampling rate.

#### Preprocessing of the Data

Accelerometer data from three orthogonal dimensions are filtered with a Butterworth bandpass (0.3–3.5 Hz) filter. At each time point, Euclidean norm of the accelerometer data vector was calculated. The resulting time-series data was down-sampled by a factor of 4. This process is done for all data collected from all patients over the 6 months period. Two representations of the data were made: (i) Binary representation: All data was pooled together and a histogram in the log scale was obtained. The resulting histogram had a bimodal distribution, one peak corresponding to higher values in acceleration and hence high activity periods, and the second peak corresponding to lower values in acceleration and hence rest periods. Taking the acceleration value of the minimum between the two peaks as a threshold, acceleration magnitudes higher than the threshold were represented by 1’s and acceleration magnitudes smaller than the threshold were represented by 0’s. (ii) Smooth representation: A median filter with length of 6 h was applied to the down-sampled data to get a smooth estimate of the activity levels.

#### Extracting Nighttime (Sleep Segment)

Individual 24-h data segments were extracted from 12:00 pm midday on a given day to the next day 12:00 pm midday. The data was labeled with the binary representation for an initial estimate of the active – 1’s and rest – 0’s periods during the given 24-h window. This window consisted of three segments: daytime (segment prior to sleep), nighttime (sleep segment), and daytime (segment after sleep). We proposed that the nighttime segment would consist of more 0’s than 1’s and daytime segments would consist of more 1’s than 0’s. Therefore, we defined an ideal nighttime model built with a function that takes a value 0 within continuous period of duration “L” centered at a time “T” with a value 1 outside this region. Given an initial estimate of L and T, the difference between the ideal nighttime model and the binary representation of movement was computed using a quadratic cost function. In this cost function each mismatch, occurring when the binary value is 1 during nighttime or 0 during daytime, contributes 1, and each match, occurring when the binary value is 0 during nighttime and 1 during daytime, contributes 0. The initial estimate for T was taken to be the time point corresponding to the minimum of the smooth representation mentioned above. Initial estimate for L is set to 8 h. We then minimized this cost function using unconstrained non-linear optimization. This led to the best model estimate for L, the nighttime length, and T, the nighttime mid-point, and allowed us to locate the borders for the three segments (daytime, nighttime, and daytime) from the 24-h window. We then extracted the nighttime segment to evaluate the micro-changes within. The optimization algorithm to find the night-time periods provides an estimate for the night-time duration and the mid-night point (see Supplementary Figure 1).

#### Identification of Rest and Active Durations During Nighttime and Relating Them to Sleep

Within the nighttime segments, periods with all 0’s is attributable to lack of movement and periods with all 1’s is attributable to movement. However, mapping these periods directly to sleep-wake periods faces the problem that the durations of these periods can range from milliseconds to hours in actigraphy data, whereas analysis of sleep is carried out by classifying non-overlapping epochs of 30 s duration into awake and asleep. To link our actigraphy analysis to the analysis timescales used in sleep studies, we took all segments of length *N* = 30 s and replaced the values in those segments by the median value over a window of 3N duration centered on the segment. In order to resolve situations that were marginal (15 s is rest and 15 s is active) it was considered reasonable to utilize a more robust estimate obtained from utilizing the adjacent windows. This is analogous to use of additional information for ambiguities in polysomnography studies. For longer duration rest periods this step should not make any difference. But for very small windows it would use the information from the neighboring windows to provide a more robust estimate of the subject state. We chose *N* = 30 s, but, found that the results were not sensitive to this exact choice. After we repeat this process for all short segments, consecutive time points in the nighttime segments corresponding to 0’s were identified as rest durations and those corresponding to 1’s were identified as active durations.

#### Determining the Distributions of Rest and Active Durations

**FIGURE 2:**
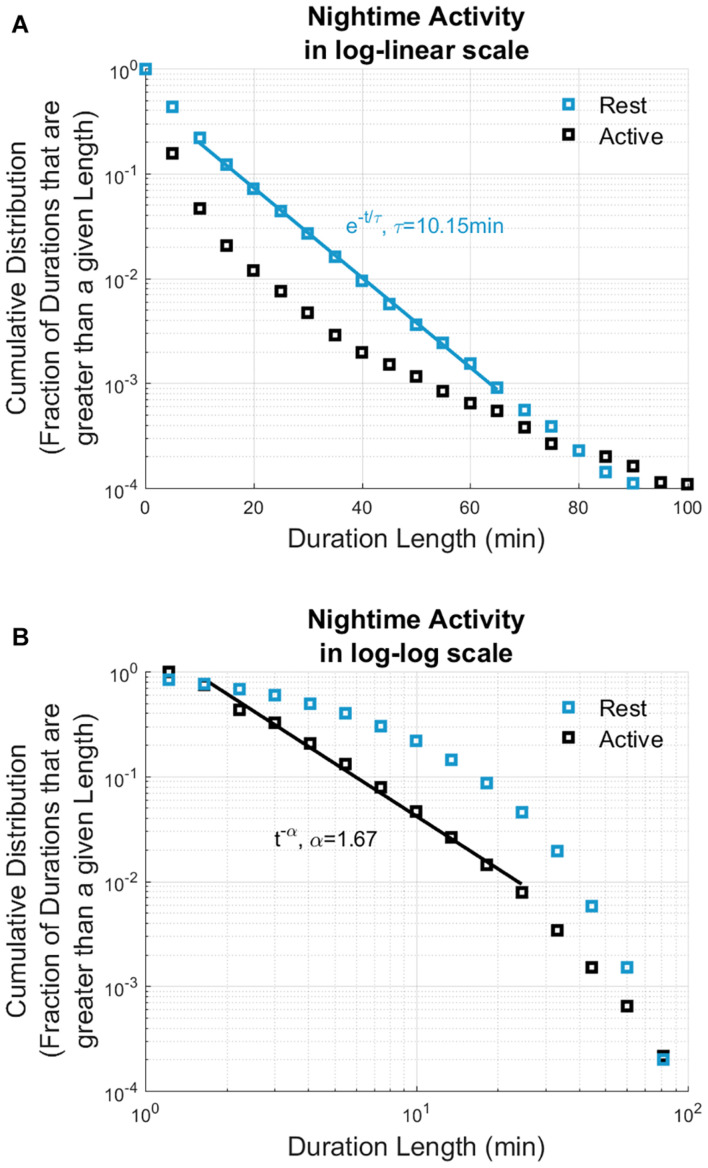
Cumulative distribution of rest and active durations based on 14,736 h of nighttime actigraphy data recorded from all participated patients (*n* = 22). The cumulative distributions of nighttime rest periods (displayed in blue) show exponential distribution, whereas active periods (displayed in black) show power law distribution. Solid lines show the best straight-line fits: rest durations can be fitted by a straight line in log-linear scale **(A)**, and active durations can be fitted with a straight line in log-log scale **(B)**. *X* axis display the nighttime durations; *Y* axis show the cumulative distributions. The current actigraphy data analysis revealed similar nighttime rest/activity dynamics to nighttime sleep/wake dynamics based on polysomnography data analysis (Lo et al., 2013).

Rest durations across all participants were pooled and the quantity $P\,\left(\text{t}\right)\equiv \int_{\text{t}}^{\infty }\text{p}\,\left(\text{w}\right)\,\text{dw}$, where p(w) is the probability density function of rest durations between w and w + dw, was examined. P(t) represents the fraction of rest durations that are greater than length t and is referred to as the cumulative distribution function. Similarly, the cumulative distribution of the active durations was also calculated, and distributions of both rest and active durations are displayed in Figure 2.

### Assessment of Functional Ability

Activities of daily living were assessed at baseline and regular monthly intervals during the 24-week treatment period in the same study population of actigraphy recordings using the clinically established ADCS-ADL scale (Galasko et al., 1997). The ADCS-ADL assesses the competence of AD patients in basic and instrumental activities of daily living. The assessments were by a caregiver in questionnaire format or administered by a healthcare professional as a structured interview with the caregiver.

### Statistics

All statistical comparisons were done using Kolmogorov–Smirnov test.

## Results

The current analysis includes results on 22 mild-to-moderate AD subjects who successfully completed the 6-month study.

### Safety

The safety profile of the treatment was benign (Table 2). Adverse events related to the study device had been reported of 42% of the participants, and 41% of the total adverse events were related to the device. Most (36%) adverse events were mild, 2.5% of them were moderate and 2.5% of them were severe. One participant in the treatment group and one participant in the sham group had discontinued the study due to an adverse event.

**TABLE 2:** Safety analysis.

Adverse events	Treatment group (*n* = 19)		Sham group (*n* = 12)	
	Patients affected n (%)	Adverse events n (%)	Patients affected n (%)	Adverse events n (%)
**Any adverse event**	13 (68)	39 (100)	10 (83)	27 (100)

**Adverse events unrelated to study device**	10 (53)	23 (59)	8 (67)	18 (67)
**Adverse events potentially related to study device**	8 (42)	16 (41)	4 (33)	9 (33)
**Mild Intensity**	7 (37)	14 (36)	4 (33)	8 (30)
Anxiety	1 (5)	1 (3)	0	0
Benign positional vertigo	1 (5)	1 (3)	0	0
Confusion	0	0	1 (8)	1 (4)
Disorientation	0	0	1 (8)	1 (4)
Dizziness	1 (5)	1 (3)	1 (8)	1 (4)
Ear Irritation	1 (5)	1 (3)	0	0
Eye irritation	2 (11)	2 (5)	0	0
Fatigue	1 (5)	1 (3)	0	0
Hallucinations	0	0	1 (8)	1 (4)
Headache	5 (26)	5 (13)	2 (17)	2 (7)
Migraines	0	0	1 (8)	1 (4)
Nose irritation	1 (5)	1 (3)	0	0
Shoulder pain	1 (5)	1 (3)	0	0
Wandering	0	0	1 (8)	1 (4)
**Moderate intensity**	1 (5)	1 (3)	1 (8)	1 (4)
Agitation	0	0	1 (8)	1 (4)
Chest irritation	1 (5)	1 (3)	0	0
**Severe intensity**	1 (5)	1 (3)	0	0
Dementia exacerbation	1 (5)	1 (3)	0	0

*Data are number of patients with an adverse event (percentage within study population; i.e., Treatment or Sham Group). Total number of adverse events (percentage of adverse events in the respective study population). Percentage values were rounded to the closest integer.*

### Adherence

We obtained the adherence rate from 21 out of 22 participants for the entire 6-month study period. The average adherence rate for these participants was 91%. For 1 participant, data was only available for the first 87 days; adherence rate for this participant was 92% during this period.

### Sleep Evaluated by Continuous Actigraphy Recordings

An average of 100 days and nights of continuous actigraphy recordings were obtained from the 22 subjects in this subgroup of patients. Data from actigraphy recordings were processed for constructing a nighttime sleep model, which allowed to assess the durations of rest and active periods during sleep. All rest and active durations identified by actigraphy data processing were pulled and analyzed from each participant as described in “Materials and Methods” section, and the results were compared to published data of rest and active periods obtained by polysomnography-based sleep analysis. As evidenced by straight-line fits on a log-linear scale, the rest durations follow an exponential distribution, *e*^−*t*/τ^with τ = 10.15*min*. In contrast, active durations follow power law distribution (straight-line fit on a log-log scale), *t*^−α^ with α = 1.67 (Figure 2). Such exponential and power law behaviors have been observed in sleep studies of healthy subjects (Lo et al., 2002, 2004, 2013). These authors analyzed nighttime sleep and awake states as obtained from polysomnographic (PSG) recordings of healthy subjects and found that cumulative distribution of sleep state durations is characterized by an exponential distribution whereas those of awake state durations were characterized with a power law distribution. Thus, the exponential decay constant as τ = 10.9*min* for light sleep, τ = 12.3*min* for deep sleep, τ = 9.9*min* for REM sleep durations, and the power law exponent as α = 1.1 for awake durations were reported (Lo et al., 2013). We also found that the nighttime rest and active durations, estimated from actigraphy recordings of AD patients show exponential and power law behavior. Similarities in the form of the distributions between our results and previous work suggest that nighttime rest and active durations as assessed by actigraphy are analogous to sleep and awake states as assessed by polysomnography and that the effect of therapy on sleep may be indirectly assessed through its effect on active and rest durations.

### Effects of Gamma Sensory Stimulation on Sleep Quality Determined by Continuous Actigraphy Recordings

**FIGURE 3:**
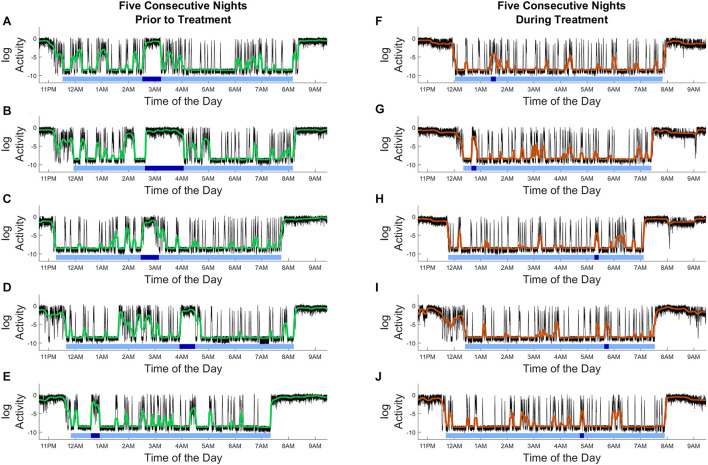
Actigraphy recordings from a single patient demonstrating the effects of gamma sensory stimulation therapy on nighttime active and rest periods. **(A–E)** Five consecutive nights prior to treatment, **(F–J)** five consecutive nights during treatment period. Level of continuous activity is shown by the black tracing; median filtered activity levels are indicated by green **(A–E)** and brown **(F–J)** curves. The applied analysis identified nighttime active and rest periods: denoted sleep periods are marked by horizontal light blue bars, and the longest movement periods during nights are indicated by the dark blue bars. Compared to the pre-treatment period, fewer and shorter movement periods are observed. *X*-axis shows the time of day, *Y*-axis shows the activity level (in log scale).

Outcomes from gamma sensory stimulation therapy on sleep were evaluated from actigraphy data which allowed to assess the durations of rest and active periods during sleep. Results from this analysis of a single patient as shown in Figure 3, demonstrate nighttime active and rest periods; the level of continuous activity is determined and indicated by the black tracing. Furthermore, intervals were identified as sleep for each night (represented by horizontal light blue bars), and the longest movement periods are indicated by the dark blue bars. Effects of gamma sensory stimulation therapy on sleep were determined by comparing the distribution of the length of nighttime uninterrupted rest durations in the first and the second 12-week periods of the study. Although patients showed a high adherence to therapy, only subjects who wore the actigraphy device for at least 50% of the time during both the first and last 12-week periods were used for assessing efficacy of gamma sensory stimulation therapy on sleep (*n* = 7 Treatment, *n* = 6 Sham).

**FIGURE 4:**
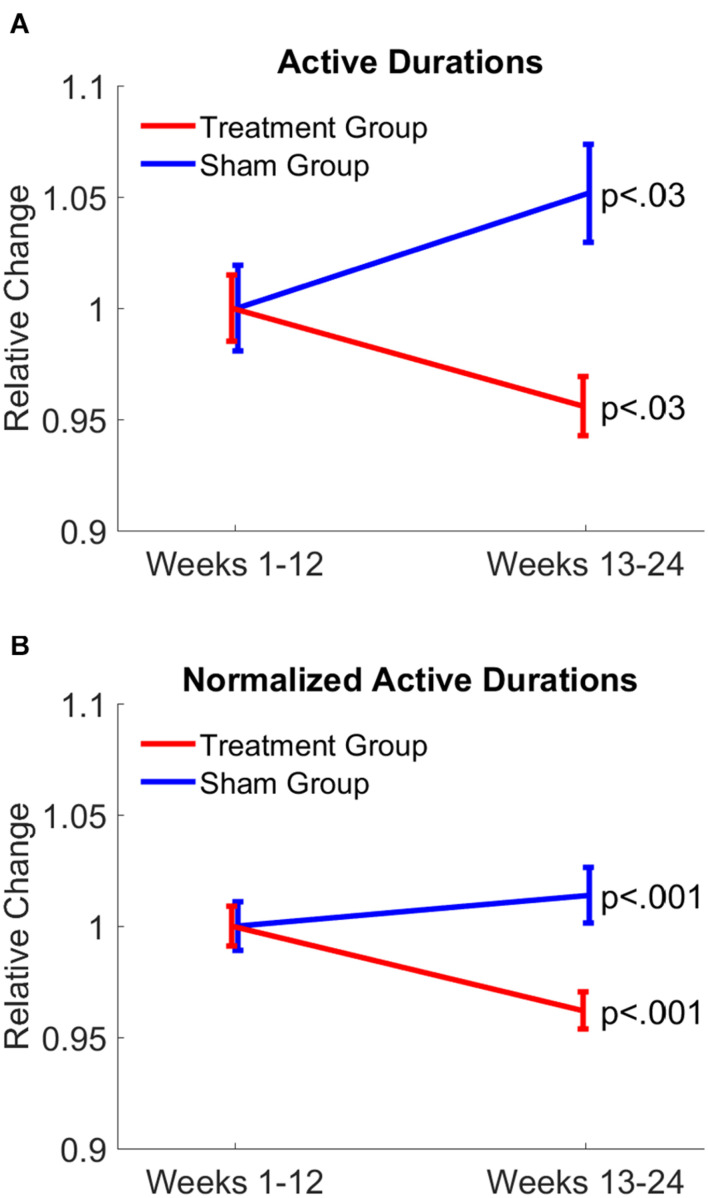
Gamma sensory stimulation therapy reduced duration of active periods during nighttime. Changes in durations of active periods in treatment group is shown in red and sham group is shown in blue from the first 12 weeks to the last 12 weeks of the therapy **(A)**. Active durations were normalized by dividing duration of each active period by the duration of the corresponding nighttime period **(B)**. Changes observed in the treatment group (red) indicate a significant reduction in duration of active periods, indicating an improvement in sleep quality, while the opposite can be assessed in the sham group (blue).

Compared to the first 12-week period, nighttime active durations were significantly (*p* < 0.03) reduced in the treatment group, whereas active durations were significantly (*p* < 0.03) increased in patients of the sham group. After active durations were normalized by dividing each active duration by the duration of the corresponding nighttime period of each patient, results further confirmed opposite changes in nighttime active durations between treatment and sham groups (*p* < 0.001, both groups; Figure 4). Furthermore, the average night-time period was 7.23 h for the treatment group, and 7.64 h for the sham group. The difference between the second 12-week period and the first 12-week period was in the order only of a couple of minutes. Specifically, the difference was −2.65 min for the treatment group and 2.07 min for the sham group. The present actigraphy findings demonstrate a reduction in nighttime active durations in response to gamma sensory stimulation therapy, leading to improvement in sleep quality, while the opposite can be assessed in the sham group.

### Functional Ability as Assessed by Alzheimer’s Disease Cooperative Study – Activities of Daily Living Was Maintained in Patients Treated With Gamma Sensory Stimulation

**FIGURE 5:**
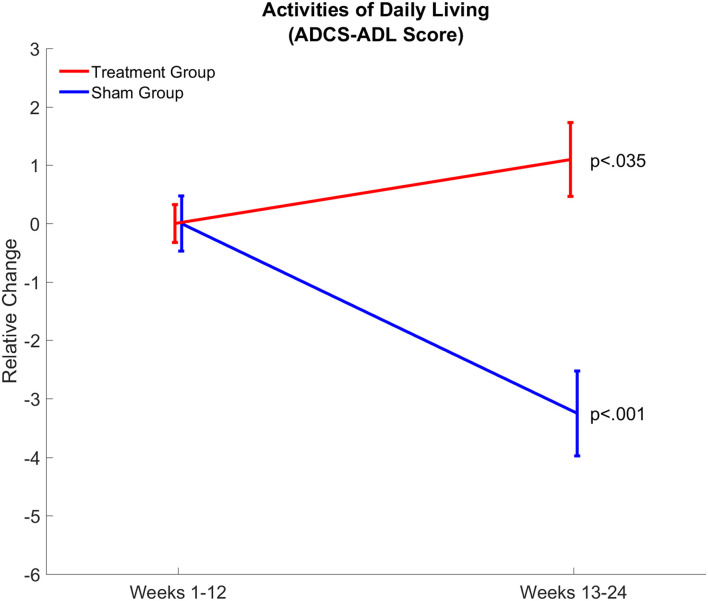
Gamma sensory stimulation therapy results in maintenance of daytime activities assessed by Activities of Daily Living (ADCS-ADL) Score. Relative changes in daytime activities are shown between weeks 1–12 and weeks 13–24 in the treatment (red; *n* = 14) and sham (blue; *n* = 8) groups. Changes in daytime activities showed a significant (*p* < 0.035) improvement in the treatment group and a significant (*p* < 0.001) decline in the sham group.

Effects of gamma sensory stimulation treatment on patients’ ability to perform activities of daily living were evaluated by comparing average ADCS-ADL scores from the first 12-week and second 12-week periods in both treatment (*n* = 14) and sham (*n* = 8) groups (Figure 5). Each patient in the sham group showed a decline in ADCS-ADL scores, resulting in a significant decline (*P* < 0.001) of approximately three points in this patient group over the trial period. In contrast, nine out of 14 patients in the treatment group maintained or improved their ADCS-ADL scores. Therefore, the average ADCS-ADL score in the treatment group significantly (*p* < 0.035) increased during the treatment period.

## Discussion

The present findings demonstrate that of 40 Hz audiovisual stimulation, eliciting 40 Hz gamma oscillation improves sleep quality and maintains functional abilities in mild to moderate AD patients and confirm a positive safety profile of the treatment with high patient adherence. The current findings are in line with a recent publication reporting that 4- or 8-weeks gamma sensory stimulation therapy is safe, tolerable, and feasible in subjects with mild cognitive impairment due to AD (He et al., 2021). The current results also provided evidence for applicability of continuous, long-term actigraphy recordings of nighttime activities in AD patients, suitable for assessing impact of potential therapeutics on sleep quality.

Sleep disorders in MCI and AD patients are well recognized, having a 35–60% prevalence of some form(s) of sleep abnormalities (Vitiello and Borson, 2001; Peter-Derex et al., 2015). Though early detection of sleep disorders has a particular significance given the established link between sleep disfunction and AD pathology, detecting these pathological changes in patients are challenging. The practicality of sleep questioners used in clinical practice for sleep disorders, such as the Pittsburgh sleep quality index or Athens insomnia scale provide limited value since patients frequently do not recognize their sleep disturbances (Most et al., 2012). Unquestionably, PSG studies are best suited to detect and monitor of sleep abnormalities, however, performing PSG recordings could be difficult for MCI or AD patients. Furthermore, monitoring sleep changes with PSG in response to therapeutic intervention over a longer period of time is impractical in MCI and AD patients. Recently, sleep monitoring with actigraphy in AD patients has become more frequent since a strong correlation between PSG and actigraphy data in sleep and wake time periods has been established (Ancoli-Israel et al., 2008). Our current findings show that wrist-worn actigraphy over 6 months is feasible and very well tolerated by participants. Longitudinal monitoring of sleep is a clear advantage when the onset of the treatment is unknown, like in our testing of gamma sensory stimulation therapy. Analysis of the current actigraphy data revealed identical nighttime rest/sleep – activity/awake dynamics to those which were based on PSG data analysis (Lo et al., 2013). This observation further validates the applicability of continuous monitoring of nighttime sleep-wake activity with actigraphy, and its suitability for AD patients.

Results, based on the collected actigraphy data over a 6-month period, demonstrate that gamma sensory stimulation therapy can significantly reduce active periods during night in mild to moderate AD patients. In contrast, patients in the sham group do not show improvement in sleep characteristics. These findings are in agreement with recent experimental finding demonstrating that a 40-Hz light stimulation alleviates abnormal circadian rhythm in Aβ overproducing transgenic APP/PS1 mice and significantly reduces abnormally high activity during their rest phase. These changes were accomplished by normalizing activity of suprachiasmatic neurons and the expression levels of proteins regulating the central circadian clock (Yao et al., 2020). The current findings demonstrating a beneficial effect of gamma sensory stimulation therapy in mild to moderate AD patients by prolonging nighttime undisturbed restful periods is considered as a translational biomarker demonstrating similar functional outcome in preclinical and clinical studies.

Pathomechanisms underlying sleep dysfunction in MCI and AD patients are mostly unknown, though AD-related pathological proteins, such as Aβ- and tau- oligomers are known to disrupt sleep but their mode of action is unknown. Brainstem ascending neurons considered playing a role in sleep-wake regulation, including cholinergic, serotoninergic and norepinephrine neurons show profound degeneration starting from an early stage of the disease (Smith et al., 2018; Tiepolt et al., 2019; Kang et al., 2020). Similarly, suprachiasmatic nucleus containing neurons playing a key role in regulating circadian rhythms also shows neurodegeneration early in the disease (Van Erum et al., 2018). Whether similar mechanisms are involved in response to 40 Hz sensory stimulation in AD-related transgenic mice and AD patients is an open question at the moment.

There are only limited treatment options for sleep abnormalities in MCI and AD patients, and pharmacological treatments options include antidepressant, antihistamines, anxiolytics, and sedative-hypnotic drugs, some of them known to impair cognitive function and interference with motor behavior (Vitiello and Borson, 2001; Deschenes and McCurry, 2009; Ooms and Ju, 2016). Recently, suvorexant, an orexin receptor antagonist has been approved as a sleep medication for AD patients having clinically diagnosed insomnia. The main effects of suvorexant are a prolonged total sleep time and delayed wake after sleep onset, without impacting sleep fragmentation or altering sleep architecture (Herring et al., 2020). In the present trial evaluating gamma sensory stimulation, clinically diagnosed sleep abnormality such as insomnia has not been an including criteria requirement, consequently beneficial effects of gamma sensory stimulation are not limited to AD patients suffering from clinically recognized sleep problems.

Beneficial effect of gamma sensory stimulation on sleep quality in mild to moderate AD patients is particularly relevant, since scientific and clinical observations demonstrating a close relationship between sleep quality and activities of daily living (Zhou et al., 2019). It can be presumed that improving sleep quality in AD patients would provide multiple benefits: better sleep will enhance patients’ daytime performance and reduce daytime sleepiness. In line with this hypothesis, patients on gamma sensory stimulation treatment maintained functional activity as reflected by their unchanged ADCS-ADL score over the 6-month treatment period. In contrast, ADCS scores of sham group patients dropped similarly to changes of placebo group patients observed previously in clinical trials (Doody et al., 2013, 2014). Even though the close relationship between sleep and daily activity is well documented, it is unknown at present whether improved sleep quality is the main factor contributing to the maintenance of ADCS-ADL scores in gamma sensory stimulation treated patients, or improvement in sleep and continuation of functional ability are unrelated positive outcomes from the therapy.

Currently, the underlying mechanisms of improved sleep and maintained functional ability of AD patients in response to gamma sensory stimulation are not known. Preclinical studies indicate that 40 Hz sensory stimulation reverses Aβ and tau pathologies, improves synaptic function leading to improved cognitive function in transgenic mice (Iaccarino et al., 2016; Adaikkan et al., 2019; Martorell et al., 2019). Although human AD-related biomarker studies are in progress, at the moment it is unknown if the same biochemical and neuroimmunology mechanisms are activated in AD patients as identified in mice. The bidirectional interaction between sleep and disease progression (Wang and Holtzman, 2020) supports the notion that improved sleep in response to gamma sensory stimulation treatment could also slow down disease progression.

## Data Availability Statement

The raw data supporting the conclusions of this article will be made available by the authors, without undue reservation.

## Ethics Statement

The studies involving human participants were reviewed and approved by the Advarra IRB Research Ethics Board. The patients/participants provided their written informed consent to participate in this study.

## Author Contributions

AC had full access to all the data in the study, takes responsibility for the integrity of the data and the accuracy of the data analysis, and contributed to analysis and conceptualization of analysis approach. ZM and EH conceived and designed the study. EH, KM, and TT executed the study. AC, MH, and ZM interpreted the data. MH and AC drafted the manuscript. ZM, EH, TT, NS, KM, and AC provided administrative, technical, or material support. All authors contributed to the article and approved the submitted version.

## Conflict of Interest

All authors were employed by Cognito Therapeutics, Inc. at the time of this study.

## Publisher’s Note

All claims expressed in this article are solely those of the authors and do not necessarily represent those of their affiliated organizations, or those of the publisher, the editors and the reviewers. Any product that may be evaluated in this article, or claim that may be made by its manufacturer, is not guaranteed or endorsed by the publisher.

## Footnotes

1
https://clinicaltrials.gov/ct2/show/NCT03556280
