# Supplementary material for: Sensory-Evoked 40-Hz Gamma Oscillation Improves Sleep and Daily Living Activities in Alzheimer’s Disease Patients
Source: Front Syst Neurosci. 2021 Sep 24;15:746859. doi: 10.3389/fnsys.2021.746859 (PMC8500065; doi:10.3389/fnsys.2021.746859)
Supplement: Supplementary file 1 [file Image_1.pdf]

Supplementary Material

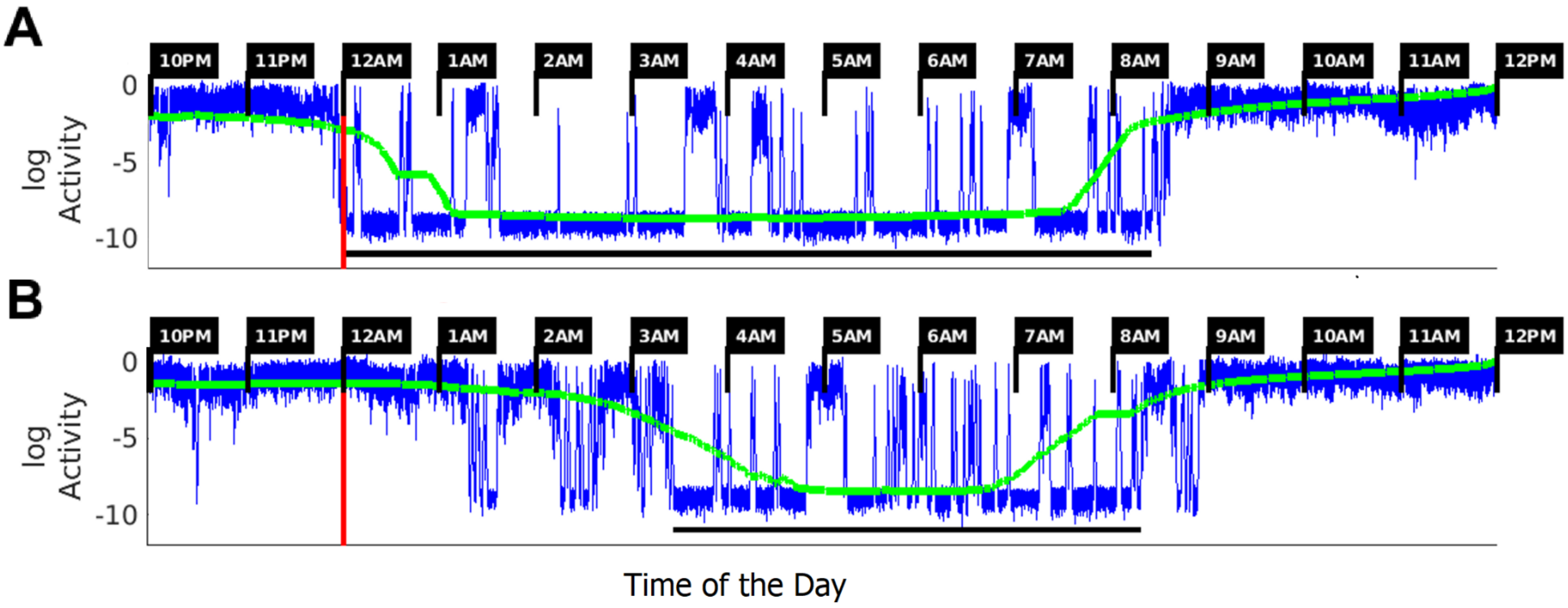

Supplementary Figure 1. Algorithm performance on two datasets with night-time periods of different lengths. Two actigraphy recordings are shown with activity in blue and a smooth representation of the activity in green. The smooth representation is obtained by using a moving median filter with 6-hour duration. The timepoint corresponding to the minimum of the smooth representation is the initial value of the mid-night point, T, for the algorithm. The initial value for the night-time duration, L, is taken to be 8 hours. These values are used as inputs to the algorithm which then determines the optimum values for T and L. 12AM is marked as a vertical line (red) for reference. Results of the optimization algorithm are shown in horizontal line (black). **A.** Optimum night-time period is ~8 hours and mid-night point is ~4AM. **B.** Optimum night-time period is ~5 hours and mid-night point is ~6AM. Note that the algorithm is successfully able to determine the night-time periods despite the differences in their lengths.
